# Late Manifestation of Invasive Adenocarcinoma in a Right-Colon Interposition for Esophageal Atresia

**DOI:** 10.7759/cureus.96257

**Published:** 2025-11-06

**Authors:** Kristina Yancey, Katherine Macmillan, Terry Jue, Kumar Sandrasegaran

**Affiliations:** 1 Radiology, Mayo Clinic, Phoenix, USA; 2 Gastroenterology, Mayo Clinic, Phoenix, USA

**Keywords:** colon adenocarcinoma, colonic interposition, esophageal atresia, esophageal reconstruction, gastrointestinal neoplasm

## Abstract

Colonic interposition is a recognized surgical technique for esophageal replacement in patients with esophageal atresia. While generally effective, this approach carries a risk of long-term complications, including the rare development of adenocarcinoma within the interposed segment.

We present the case of a 59-year-old man with a history of esophageal atresia repaired in infancy using a right-sided colonic interposition. He presented for evaluation due to worsening dysphagia and early satiety. Endoscopic examination revealed a 20 mm ulcerated lesion at the distal anastomosis, and biopsy confirmed intramucosal adenocarcinoma. Fluoroscopic esophagram and PET-CT demonstrated a mass-like lesion with focal hypermetabolism but no evidence of metastatic disease. Given significant surgical risk due to prior abdominal surgeries and poor nutritional status, the patient elected to pursue systemic immunotherapy with close imaging and endoscopic surveillance.

Adenocarcinoma arising in colonic interpositions is exceedingly rare, with latency periods typically ranging from 20 to 40 years. In this case, malignancy developed nearly six decades after the initial repair. This case highlights the potential for malignant transformation many decades after esophageal reconstruction and underscores the importance of sustained clinical follow-up and endoscopic surveillance in patients with colonic interpositions.

## Introduction

Esophageal atresia is a congenital anomaly characterized by discontinuity of the esophagus, requiring early surgical intervention to restore alimentary continuity. When primary anastomosis is not feasible - such as in long-gap atresia or after failed repair - surgeons may reconstruct the esophagus using an interposed segment of stomach, jejunum, or colon. Colonic interposition, in particular, provides a durable conduit between the pharynx and stomach, maintaining swallowing function and allowing for long-term nutritional support.

Among colonic interpositions, left-sided grafts are generally preferred because they offer reliable perfusion and a wide-diameter lumen [1,2]. Despite favorable long-term outcomes, late complications such as anastomotic stricture, redundancy, dysmotility, and gastrocolic reflux have been reported. A rare but serious complication is malignant transformation of the interposed colonic segment [3-6].

We report a case of adenomatous polyps and adenocarcinoma arising in a right-sided colonic interposition nearly 60 years after post-neonatal esophageal atresia repair. This case underscores the importance of endoscopic and imaging surveillance for early detection of malignancy in patients with long-standing colonic interpositions.

## Case presentation

A 59-year-old male with a history of esophageal atresia initially presented for evaluation of progressive intermittent dysphagia. He was diagnosed with esophageal atresia at birth. He had a feeding tube his first year of life before undergoing retrosternal right-sided colonic interposition at age one. The exact surgical details are unavailable, but he describes what may have been an ‘End to Side’ anastomosis. The surgery was successful, allowing a transition to oral feeding.

Throughout adolescence and adulthood, he experienced chronic reflux symptoms. In early adulthood, he was diagnosed with gastric ulcers and Helicobacter pylori infection, which were treated successfully.

At age 49, he underwent a laparoscopic cholecystectomy for gallstones, complicated by extensive intra-abdominal adhesions. Shortly thereafter, he developed a small bowel obstruction requiring bowel resection.

At age 59, he developed progressive dysphagia to solid foods and early satiety, though he could still tolerate liquids. He did not report significant weight loss, vomiting, or aspiration. An esophagogastroduodenoscopy (EGD) at an outside institution revealed multiple large diverticula within the interposed colon and a 20 mm cratered ulcer at the distal colonic anastomosis, which was biopsied. The stomach contained multiple sessile polyps, but due to his tortuous anatomy, the pylorus and duodenum were not visualized. Pathology from the ulcer revealed intramucosal adenocarcinoma.

**Figure 1:**
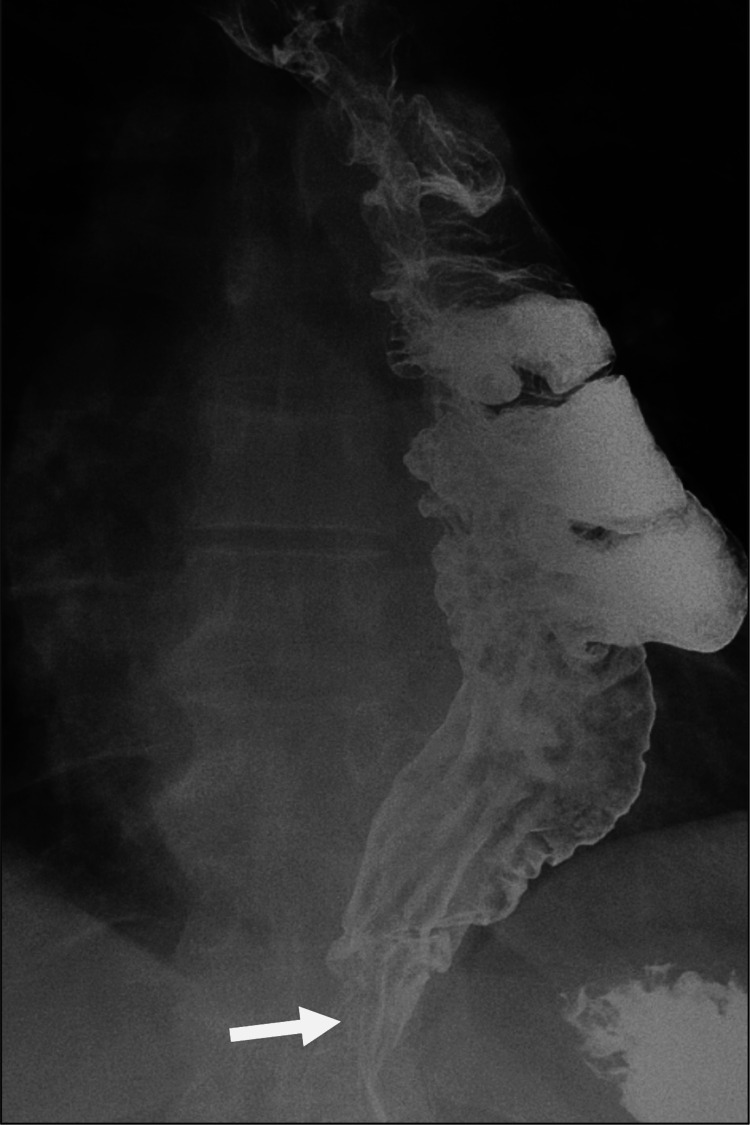
Mass-like shelf at the distal colonic interposition. Single-contrast barium esophagram shows a shelf-like filling defect (arrow) at the lower colonic interposition, suspicious for neoplasm.

**Figure 2:**
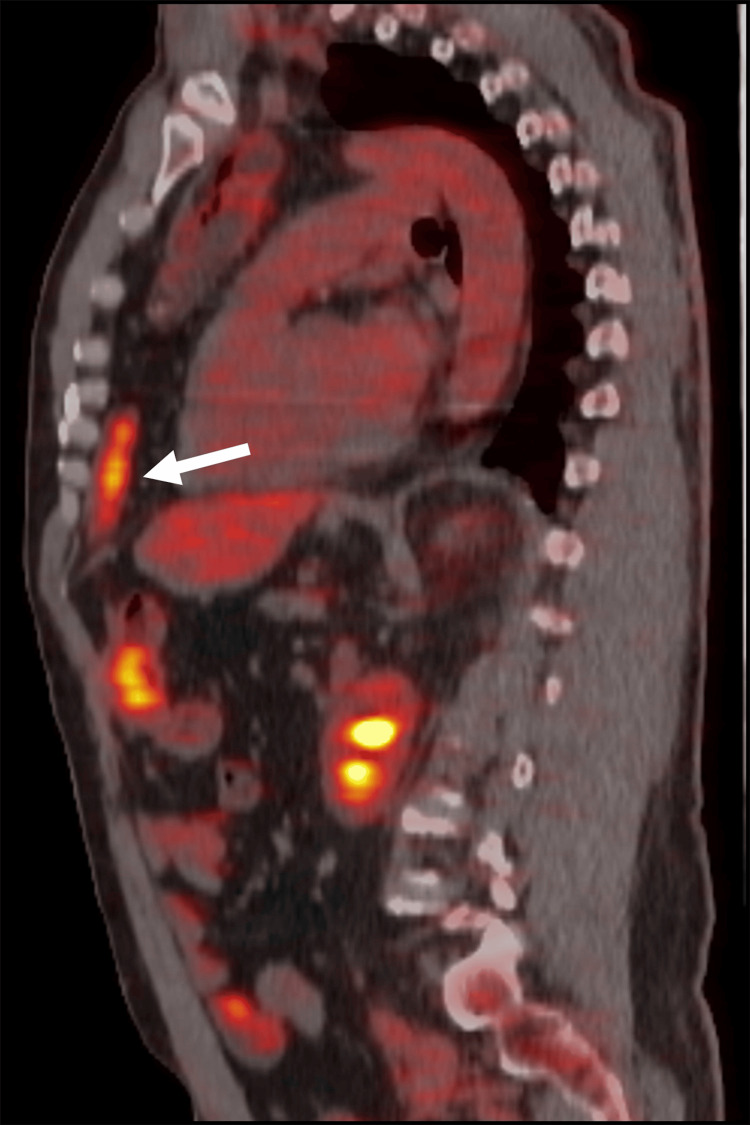
FDG-avid lesion in the distal colonic interposition. 18-fluorodeoxyglucose (FDG) PET/CT demonstrates focal hypermetabolism (arrow) in the distal colonic interposition, consistent with tumor involvement.

After referral to our institution, a fluoroscopic esophagram revealed an irregular shelf-like defect in the right side of the distal interposed colon (Figure 1), concerning for cancer. A subsequent PET-CT scan revealed hypermetabolism at this site, but no evidence of metastatic disease (Figure 2). 

**Figure 3:**
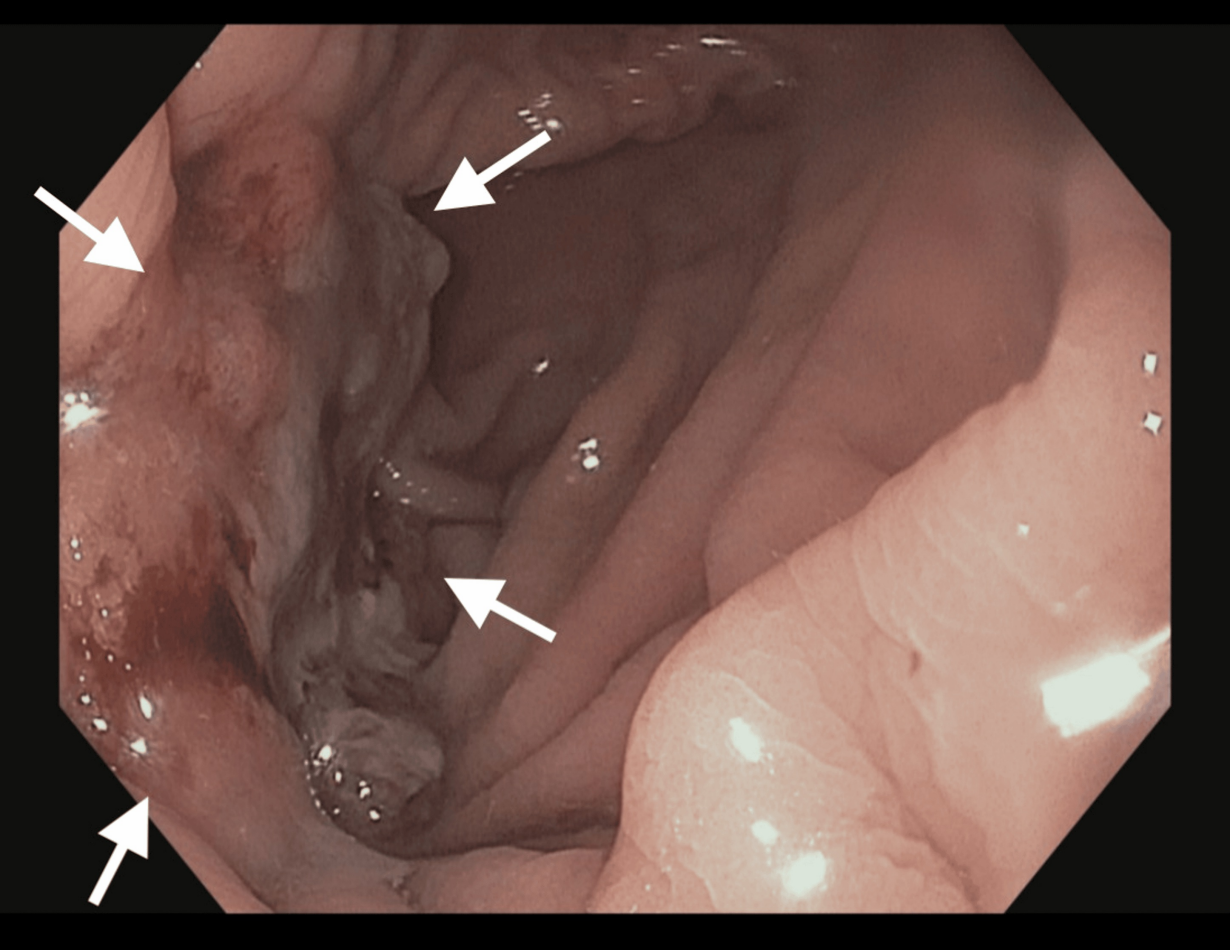
Ulcerated lesion in the distal colonic interposition. Esophagogastroduodenoscopy (EGD) image shows an ulcerated mass (arrows) with mucosal fullness and retracted colonic folds.

**Figure 4:**
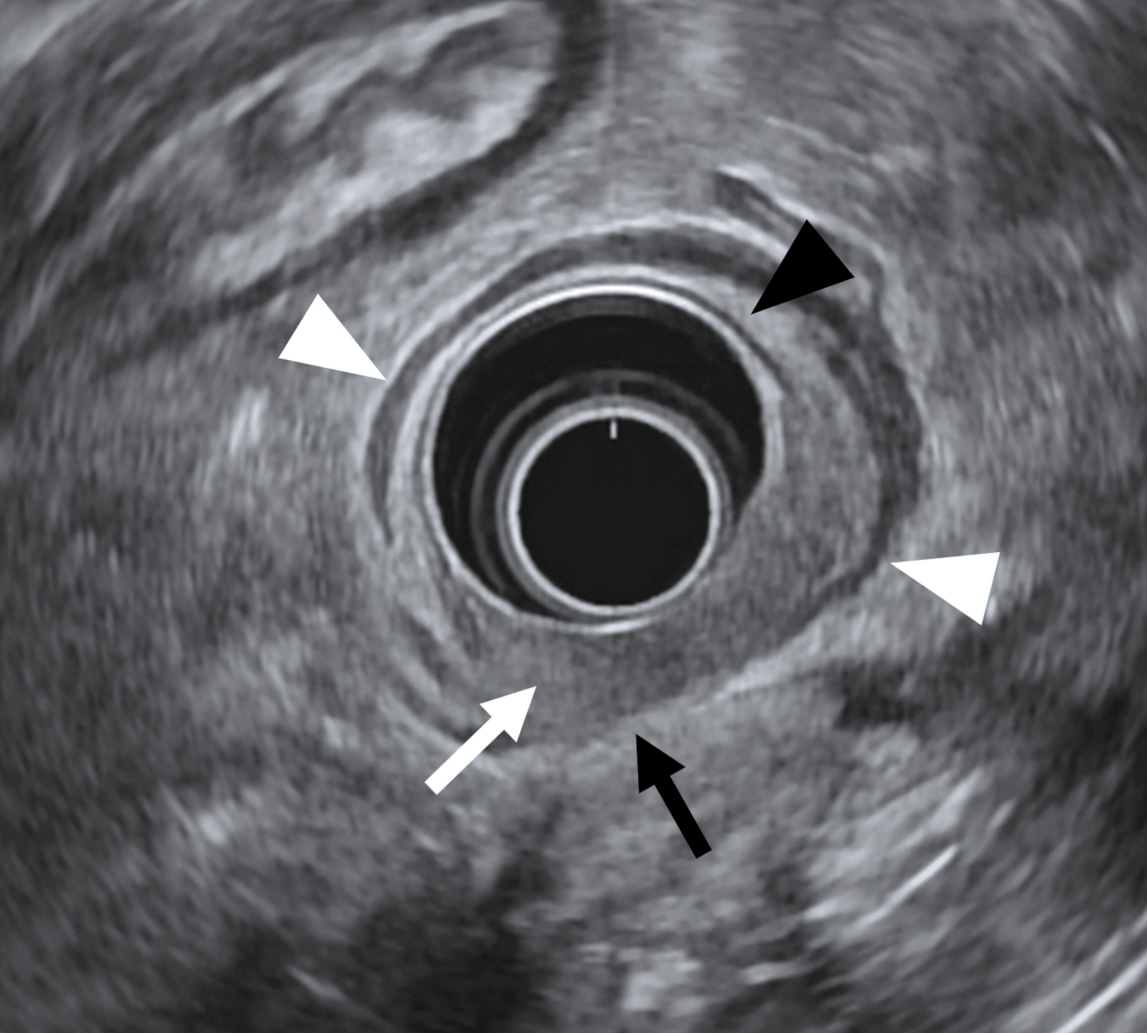
Endoscopic ultrasound of colonic mass with muscularis invasion. Endoscopic ultrasound shows a hypoechoic colonic mass (white arrow) extending into the muscularis propria. The normal hyperechoic submucosa (black arrowhead) is preserved anteriorly, while the muscularis propria (white arrowheads) is disrupted posteriorly (black arrow), consistent with tumor invasion.

The patient underwent esophagogastroduodenoscopy (Figure 3) and endoscopic ultrasound (Figure 4), which revealed an ulcerated mass in the distal colonic interposition with mucosal fullness and retracted folds. 

**Figure 5:**
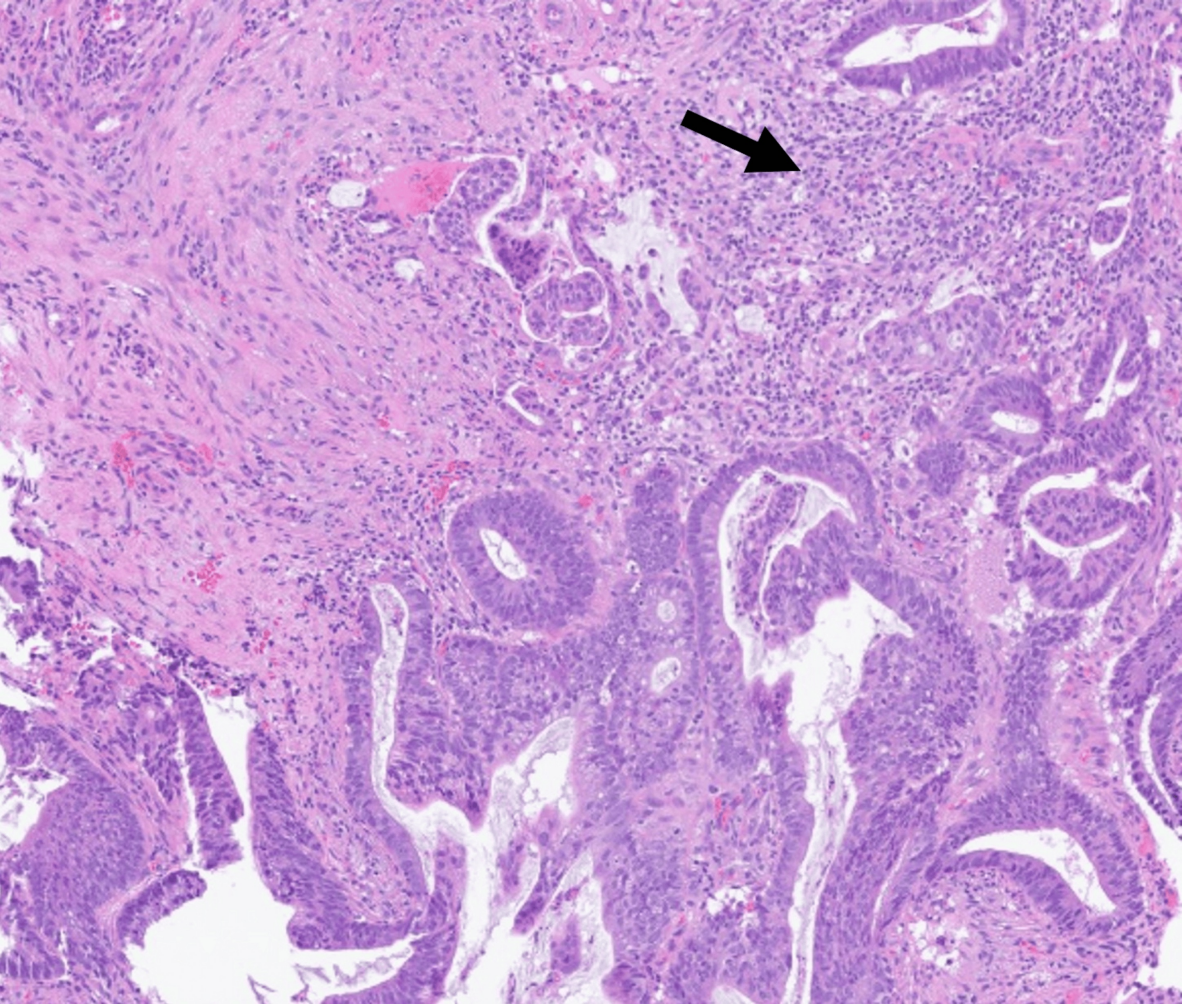
Histopathology of colonic interposition biopsy showing invasive adenocarcinoma. Hematoxylin–eosin–stained section (×40) demonstrates moderately differentiated adenocarcinoma with submucosal tumor extension (arrow), consistent with pathologic stage II (pT3N0M0) disease.

Biopsies were taken and histopathologic examination revealed a moderately differentiated invasive intestinal-type adenocarcinoma arising within the colonic interposition. The tumor extended into the subserosa without lymphovascular invasion, consistent with pathologic stage II (pT3N0M0) disease (Figure 5). 

Given the complexity of his condition and the rarity of malignancy in a colonic interposition, the patient was presented with multiple surgical options, including resection of the colonic interposition with gastric pull-up reconstruction. However, because of the significant perioperative and postoperative risks, the patient elected to pursue systemic therapy.

In light of these considerations, non-surgical treatment options were explored. Although systemic cytotoxic chemotherapy is the standard first-line therapy for advanced colorectal-type adenocarcinoma, the patient’s deficient mismatch repair/microsatellite instability-high (dMMR/MSI-H) status made him a candidate for immune checkpoint inhibitor therapy. Given the demonstrated clinical benefit of immunotherapy in dMMR/MSI-H malignancies, pembrolizumab was initiated every three weeks, with the option to transition to every six weeks, for a planned duration of up to two years.

The patient remains under active treatment with interval endoscopic and imaging surveillance every four months to assess therapeutic response and monitor for disease progression.

## Discussion

Colonic interposition for esophageal replacement has been performed for several decades. In contemporary pediatric surgical practice, the left colon is favored for esophageal replacement in congenital atresia due to its reliable blood supply (via the inferior mesenteric artery), more compatible luminal diameter, and relative ease of mobilization [1,2,7]. In our subject, the right colon was used for replacement, for reasons that are unclear. Detailed operative records of the original procedure were unavailable, which limited further insight into the surgical approach, vascular supply, and anastomotic configuration. 

Colonic interpositions have also been used following esophageal cancer resection when the stomach is unsuitable for a pull-through procedure. Immediate postoperative complications include pneumonia, ischemic colitis, chylothorax and anastomotic leak or stricture [8,9]. Over time, intrathoracic colonic segments can develop marked tortuosity, a phenomenon that is notably prevalent in substernal right colon interpositions [2], as was performed in our case. Patients may have delayed transit as a result and they are predisposed to regurgitation and aspiration [7,10]. 

Long-term complications in patients who underwent colonic interposition as infants include bezoar formation, dumping syndrome, gastrocolic reflux, bacterial overgrowth, kyphoscoliosis, restrictive lung disease and delayed puberty [7]. The risk of Barrett’s-type inflammation in the interposed colon was found in 7% of a cohort of 271 patients with esophageal atresia and colonic replacement [11], with a median age of diagnosis of 32 years. 

Adenocarcinoma in an interposed colonic segment is a rare but serious complication. The exact pathogenesis is unclear, but proposed mechanisms include chronic exposure to gastric and bile acids, as well as microbiome alterations. Most cases are diagnosed 20 to 40 years postoperatively [3-6,11]. 

Our patient had no routine endoscopic surveillance and presented with cancer nearly 60 years after colonic interposition surgery. This underscores the importance of lifelong surveillance in individuals with colonic interposition [7]. Current literature suggests that endoscopic monitoring should begin as early as age 20 [11]. Early detection allows for complete surgical resection, which remains the best curative option [12]. Another issue highlighted by our case is that patients who undergo colonic interposition would benefit from longitudinal care at specialized centers with interdisciplinary support. This would prevent delays in diagnosis and treatment.

## Conclusions

This case underscores the potential for malignant transformation within long-standing colonic interpositions after esophageal atresia repair - a rare but clinically significant complication. Lifelong vigilance through endoscopic and imaging surveillance remains key to early detection. Beyond individual patient management, this case also highlights the importance of awareness among clinicians caring for adults with a history of childhood esophageal reconstruction, as timely identification of premalignant or malignant changes can substantially improve outcomes.
